# Pathological Observations.—Part II. On Continued Fever, Ague, Tic Doloureux, Measles, Small Pox, and Dropsy, &c.

**Published:** 1829-10-01

**Authors:** 


### Pathological Observations, Part II, on Continued Fever, Ague, Tic Doloreux, Measles, Small Pox, and Dropsy, &c.; with a preliminary Dissertation on the Institutes of Medicine. By *William Stoker*, M.D., &c. Dublin, 1829.

Dr. Stoker has been so long known to the medical world, that neither he nor his works require any formal introduction. His rank and length of service, as a public officer, in one of the largest establishments for disease in the British Empire, and his various writings upon various subjects have obtained for him an honourable and well-merited notoriety. His pathological labours, in particular, have excited considerable notice, and the efforts which he has made to re-establish the old, and nearly obsolete doctrines of the Boerhaavian school, have characterized him as an original and reflecting physician. These efforts, however, seem hitherto to have been more highly estimated upon the Continent than with us,—in Germany, especially, they have partially succeeded; yet, although the school of Cullen is still strong, he who attentively observes the signs of the times, cannot fail to perceive that the influence of the Scotch Nosologist is considerably on the wane.

That the humoral pathology ought never to *preside* in medicine we are convinced; that it never shall again, we believe; but we have ever thought, and do still think, that the doctrines of Solidism have cast it too far back,—into *unmerited* neglect, and that while it must be admitted that brain, and bone, and every other solid texture of the body have diseases peculiar to themselves, apart from the fluids, it would be more than difficult to prove that the latter are entirely exempt from derangement. How far this derangement may obtain, and how frequently it may occur, are questions more easily started than replied to; for he, who feels it no task to expose the ravings of Bontekoe, may have some trouble in establishing the utter groundlessness of principles, which he has extravagantly over-rated and foolishly misapplied. Certain it is that, however beneficial Solidism has been to the general advancement of medical science, it has unquestionably led us into a most unwarrantable neglect, as to the chemistry of healthy and diseased animal fluids. Were Doctor Stoker's writings, therefore, of no other use than to rectify us upon this point, they would be valuable; and since pathology is the *spoiled child* of the day, we trust that more attention shall hereafter be devoted to the alterations, whether primary or secondary, occasioned by disease within the humoral department of our system, and that a portion of the labour, which is now spent, perhaps wasted, in delineating the most shadowy aberrations of the solids from healthy structure, will henceforward be given to the study of humoral pathology.

It would be both pleasing and profitable to enter into the Doctor's entire system, and endeavour to ascertain how far it is applicable to the present state of disease; but as this is an undertaking too extensive for our pages, we must restrict our review, *pro tempore*, to a single branch of the subject. Without, therefore, noticing at greater length the *Preliminary Dissertation on the Institutes of Medicine* which prefaces the *Medical Report*, we shall at once take up the Report itself, and examine with some attention the applicability of the doctrines developed in the Dissertation to the nature and treatment of *Fever.*

It appears that from the year 1804 to the close of 1827, fever had been increasing both in extent and severity in the Irish metropolis; that, in 1828, it very remarkably declined; and that, during all this period, disease and crime were strikingly proportional. In 1804 the number of fever cases received into the Cork Street Hospital was only 415; in 1810 the admissions increased to 1774; in 1815 to 3789; in 1818 to 7608; in 1827 to 6544; and in 1828 they fell to 2964; so that the proportion between the admissions of 1804 and 1827 is nearly as 1 to 16, or, holding the tables out of which these results are taken to be correct, and the Cork Street Hospital to be a fair criterion whereby to judge of the state of fever in Dublin, this disease has in the short space of twenty three years multiplied sixteen fold in that city. But, when we are informed that the Cork Street Hospital was the only asylum for fever in Dublin in 1804, and that, in consequence of its increasing prevalence, similar Hospitals were subsequently erected, the above *status* must fall considerably short of the truth, seeing that the registry of Cork Street is descriptive of the state of fever in only one section of the town. The causes of such an alarming multiplication of disease, in so short a period and over so limited a space, constitutes a very interesting subject of enquiry, and the researches of the author have certainly furnished us with a partial explanation. From 1804 to 1818 the condition of the poor, in many parts of Ireland, was unmixed wretchedness. Destitute of the necessaries of life, they became weak and enervated; dispirited by the miseries which surrounded them, every habit of cleanliness and order was neglected; filth and famine thus conspired to the propagation of disease, and constitutions enfeebled by misery and want became easy victims to a malady whose favourite haunt is the hovels of the destitute. To such a state of things may partly be ascribed the gradual spread of fever during the first fourteen years of the above period of increase; but it is more difficult to ascertain the reason why, in the years 1818—26 and 27, this *modicus passus* should have been so largely over-stepped. By many it was ascribed to the increased activity of contagion, and by some to a peculiar condition of the atmosphere; while others rested satisfied with the general causes above given. We have many reasons, however, for believing that the epidemic, which prevailed in Ireland in 1818, was a very different disease from common continued fever, as well in its symptoms and course, as treatment and terminations. It prevailed almost exclusively among the poor, for when found among the rich it could generally be traced to some hovel in the neighbourhood, symptoms of extreme debility marked its progress, the remedies most effectual for its relief were nourishing diet and moderate stimuli, the appetite, in place of being lost, was frequently voracious, the skin was cool, the thirst was moderate, and scarcely any thing like genuine febrile phenomena were perceptible. The disease, in fact, was rather negative than positive; induced by hunger it was cured by diet, and propagated by filth it was alleviated by cleanliness. The countenance was care-worn and haggard, the surface pale and parched, the entire body nerveless and emaciated, and the lower extremities very frequently œdematous. The spirits were sunk, the pulse was feeble, the strength was tottering, the appetite was keen. If timely attention were obtained these *negative* symptoms soon vanished under the use of a judicious regimen, and no unusual sequelæ to their course, when they had been long neglected, was general anasarca, or some local dropsy.

It must be obvious, therefore, to any one who attentively compares, or rather contrasts these morbid phenomena with those peculiar to specific fever, that much of what has been called the *low typhus* and *epidemic fever* of Ireland merits no such appellations, that in its symptoms, progress and treatment it is essentially different, and that, arising from want and wretchedness, it called for less therapeutical than dietetical treatment. In this way it is possible to account for the exceeding prevalency of what has been styled fever in Dublin, and also for the Brunonian views with which many of her physicians have been so deeply inoculated. To bleed in such a disease would undoubtedly be death, to purge even would be dangerous, perhaps fatal. If we could believe that ever John Brown's system was calculated for good it would be here, yet even in such cases we should look with more confidence to the *Cook's Oracle* than to the *Novum Systema.* But let us not be misunderstood;—while we believe that starvation has been not unfrequently the cause of disease and the disease itself, it is unquestionable that genuine fever is often produced and propagated by want, and that the peculiarities of the producing cause are worn by the disease produced. Hence may it be possible for an experienced eye to discover the nature of the cause in the character of the symptoms, to distinguish fever arising from contagion from fever produced by malaria, and thus not merely to ascertain the origin, but to predict the progress and to regulate the treatment by an attentive study of the physical signs of the disease. In this point it gives us pleasure to be supported by Dr. Stoker.—

"The distinctness of the symptoms arising from distinct causes of Fever, may be further illustrated by the fact, that the nursetenders of the Hospital, on receiving successive patients, and in contagious Fever, from the same family are able, by describing the preceding cases, accurately to foretel a similar train of symptoms. Of the distinct natures of Fever from contagion, and from malaria, another illustration is afforded by the preceding Table. It may be perceived, that in those years when from contagion Typhus Fever was most prevalent, agues were little or not at all known; whilst in other years, as in 1809 and 1828—in which the subsidence of continued Fever was most remarkable—the frequency of intermittents was also most remarkable. I am the more desirous to dwell on these distinctions, from being persuaded that the great discrepancy of opinion which has for some time existed among the Physicians of Great Britain and Ireland, on the subject of contagion and malaria, has arisen chiefly from want of due attention to the effects of those agents separately, and also from confounding them with those from other causes. To that confusion, probably, may be attributed the many pernicious mistakes which have taken place, both in devising means of prevention and in prescribing modes of treatment for febrile diseases." 19.

It appears to the Doctor, that the type of continued fever has of late years been very considerably changed; and that that clause of Cullen's definition "paroxysmis quovis die binis," is by no means so expressively correct at present as when it was written; that, in short, fever is a more *continued* disease now than it was formerly. In this remark we entirely concur. When we look into the works of Mead, Huxham, Sydenham, and even more modern authors, scarcely any thing which can be called, in strict language, CONTINUED FEVER, is to be met with. The morning and evening exacerbations are laid down as two diurnal epochs of constant and leading importance; the intermediate time, if not apyrexial, is to be considered as occupied by symptoms comparatively mild; and much of the description would seem more expressive of remittent than continued fever. Now, however, we have the author's testimony that such is not the present type in Dublin; and, in London, we can bear witness to the very rare occurrence of remittent fever, and to the very imperfect and often imperceptible remissions which are supposed to occur in continued fever. The cause of this modified constitution is obscure, but it is probable that the great decrease of malaria, which a more enlightened husbandry and more effectual drainage have produced, may have partly occasioned it; and that an increasingly crowded population, attended, as it ever is, with filth and poverty, may have increased the virulency of contagious poison, by which the febrile type has degenerated into a more confirmed character. It is certain, that such cases as are traceable to contagion are much more uniformly severe than those which have originated from malaria; that the typhous state appears earlier and is more developed; that the disease, in place of being regularly checkered by periodical alternations of relief, is, as it were, one continued exacerbation; that the proportion of nervous to inflammatory symptoms is much increased; and the chances of a rapid or even ultimate recovery are materially diminished. These facts are of great weight, and should never be lost sight of in the treatment of this disease; for, if properly understood, they will form a most useful guide to the choice, application and continuance of our remedies. All generalizations in medicine are dangerous, because they depend upon combinations which may be hourly unhinged; but were we inclined to hazard one, we should say that continued fever, arising from what is popularly called contagion, and continued fever generated by vegetable malaria, are two very different forms of the same disease; and not more different in their constitution than they should be in their treatment. Continued fever, arising from the latter cause, (malaria,) is the connecting link between remittent fever (if there be such a fever in cold climates) and continued fever arising from the former cause (contagion.) There is difference enough in nature for establishing this distinction; and the practical tendency of the distinction is very obvious. This difference is, to an experienced observer, generally perceptible, and often striking; and we could shew, by many examples, that a fever, which *proximately* arises from contagion, but *remotely* from malaria, and *a fortiori* a fever originating *directly* from malaria, wears in general a milder character than such as, first and last, proceeds from contagion alone; so that the type of fever being thus moulded by its cause, we may have every variety of shade from synocha to typhus gravior, according as contagion and malaria operate separately, or in different doses of admixture.

These observations will partly account for the distracted state of the public creed as to the nature of fever, and for the opposing systems which are daily issuing from the press respecting its treatment. As the subject at present stands, we hold it to be nearly impossible for the medical tyro, who knows nothing of this class of diseases from actual observation, to acquire any thing like a proper knowledge of it from books. There are nearly as many schools of fever as there are of theology; and he, who comes out charged with the doxy of one school, comes out as bigotted to his system, and as prejudiced against those of his neighbours as was ever any religionist, who believed salvation to be circumscribed within the limits of his own church. Fever is nothing but debility, teaches one school; fever is nothing but inflammation, inculcates another; fever is a morbid state of the brain, says one theorist; fever is a morbid state of the intestines says another; one is giving wine while another is drawing blood; and some, (who will be considered by many as by no means the least rational,) either disgusted by such empiricism, or deterred by such opposition, leave Nature to herself, declining to interfere with a disease, which can either be cured by every mode of treatment, or can resist the bad effects of all. One epidemic is regarded as sufficient *materiel* for observation for one author; and the principles and practice, which he found adapted to it, he incautiously extends to all; another epidemic appears of so different a type, that neither these principles nor this practice will apply;—a new theory is manufactured to explain new appearances; new indications of treatment are laid down; and the old system is discarded as unfounded and deceitful! Facts geographically local, and deductions essentially partial, are thus installed into the rank of general and abstract truths;—a few meagre features, gathered out of one year's epidemic, are spread over canvass large enough for an entire portrait; and what a cautious philosophy would still have held and taught as local facts and partial inferences, inexperienced presumption generalizes into dogmata, which are all and always to be indiscriminately applied. Such an untamed spirit of generalization should be reprobated wherever it appears. It has been the upas-tree of medicine, which neither bears wholesome fruit itself, nor permits any thing to flourish within its influence;—the dead weight of our profession, under which she has groaned for so many centuries.

"Common epidemic fever, especially when contagious, as I have frequently asserted when speaking of its pathology and treatment, has not appeared *to me* at any time to be essentially inflammatory."

Such a statement as this is legitimate, because it is strictly personal; and had Dr. Stoker rested satisfied with publishing the result of *his own* experience, attention and respect would have constituted our duty. But, anxious to see the fever of London wearing the same physiognomy, and belonging to the same family with that of the Irish metropolis, we are favoured with the following passage:—

"The peculiarities of the indigenous Typhus Fever of this city, when compared with the forms under which epidemics have been described formerly in this country, or even more recently in Great Britain, would, however, in no circumstances appear more remarkable, than with respect to the temperature of the bodies of patients labouring under common Fever. For, I have found, that in a large proportion of the cases of unmixed Typhus Fever, that I have met with, the temperature of the surface of the body, was generally rather under than over the natural standard. This circumstance of diminished temperature was adverted to by Dr. Bateman to demonstrate the remarkable difference in the character of the epidemic he described, when compared with the Fevers treated by Dr. Currie; and I regret, that from various causes, I cannot show by reports kept in former years, the thermometrical admeasurement of the temperature of the skin, in the most exquisite form of Typhus Fever, as I am of opinion it would not only prove the great difference in their nature from those treated by Dr. Currie, or even by Dr. Bateman himself, but also why cold affusion has not been at any time found so beneficial in our hospital, as it was stated to be in Dr. Currie's Reports of that Remedy. Under the head of cold or tepid affusion, as ranged amongst the remedies for Fever in the succeeding pages, a Table may be seen of the temperature, in 34 cases of Fever.

"From later publications, however, on the epidemic Fevers of London, it would appear, that the differences between its characteristics there, and those we have been describing here for so many years, are not now so great as when Dr. Bateman wrote his succinct account; for, not only greater similarity than formerly appears between the symptoms and treatment, but also a greater proximity in our views of the pathology of Fever, as described in recent publications, compared with the descriptions of the 'epidemic by the Irish writers.' The same essential denominations also, founded on the same pathological views, are employed, though apparently unawares, if that is to be inferred from the total silence kept respecting any precedent for their adoption. But, however accidental this want of recognition might have been on the other side of the channel, it is surely lawful to claim our '*disjecta membra;*' with respect to Adynamic Fever more particularly, a denomination for Typhus Fever, which I shall employ, as I have hitherto done to express not only 'the putrid or malignant Fever of Sydenham; the slow nervous Fever of Huxham; the nervous Fever, of common language: the synochus, typhus mitior and gravior, of Cullen; the jail and hospital Fever; the *fièvres essentielles* of the French; the epidemic of the Irish writers; the contagious of Bateman; the typhus of Dr. Amstrong; and the proper idiopathic, or essential Fever of Doctor Clutterbuck.' Whether it exists separately and independently; or is combined with any of the other forms of febrile disease, sporadic or symptomatic. 35.

What, or who these *disjecta membra* are, we feel some difficulty in imagining. Certain it is that neither Bateman, nor Armstrong, nor Clutterbuck, nor Wilson Philip are entitled to a place among them, and although some kindred may be claimed by Dr. Stoker with Dr. Burne, the "TREATISE ON ADYNAMIC FEVER" is nothing but a description of *one form* of the disease, and, as far as we know, has been regarded by very few in any other light; and were we called upon to select one of the most flagrant breaches of nosological accuracy with which we are acquainted, the concluding part of the above quotation should be fixed upon.* It would not be less philosophic nor practical, we maintain, to group into one description, and to subject to the same treatment, every species of inflammation, whether phlegmonoid, rheumatic, arthritic, scrofulous, puerperal, or erysipelatous, than to identify the disease described by Clutterbuck with the typhus gravior of Cullen, or to lay down curative indications for the management of Bateman's fever, which would equally apply to that of Sydenham. If debility be the characteristic of fever in Dublin, it is certainly not so in London, for the lancet is, perhaps, more freely and more favourably employed here, in the present day, than it was in those of Bateman; and it is a fact, which is not more singular than true, and which the author's assertion leaves us in some difficulty to account for, that the Irish, who are in London, are just that class of patients who require and withstand the most active measures. We believe they are the strongest *birds of passage* which we have. Robust in body and spirited in mind, unrelaxed by luxury and hardened by exercise, disease of whatever kind readily assumes in them an active character, and, in fever, during the stage of excitement, their organs are obnoxious to an unusual proportion of inflammatory attacks. Why

* We are sorry to say that grammar, as well as nosology, is violated. We beg Dr. Stoker to look at the last sentence, between the period after the word "CLUTTERBUCK," and the period after "SYMPTOMATIC," and say what is its meaning, or by what grammatical rule it is constructed.—*Ed.*

there should exist such a difference between the Irish constitution in London and Dublin, as that, during the self-same disease, it must be supported by wine in the one metropolis, and reduced by bleeding in the other, Dr. Stoker may, probably, but we cannot explain.

Let the Doctor, therefore, not be deceived by imagining, nor deceive others by asserting, that the London fever has so far degenerated as to require bark and wine in place of bleeding and purgatives; for we can assure him, and we speak advisedly, that Bateman's "Succinct Account" is almost, if not altogether, as applicable to 1829 as it was to 1818; and that those few among us, who may think otherwise, are either the remaining children of the old school, or have been educated in the new under unfavourable circumstances. We even feel a violent inclination to suspect from the writings of Dr. Mills and others, that Bateman could yet be read with some advantage in Dublin; that Armstrong might yet practise among its inhabitants without much danger; and that the degeneration, which our practice now and our politics lately have been taxed with, is not much more extensive among ourselves than among our neighbours. Dr. Stoker is, however, an experienced and observing physician, and we shall proceed to present our readers with a condensed abstract of his views on the more important points of this interesting subject.

After dividing all fevers into inflammatory, typhoid and intermittent, and noticing his intention to "arrange the cases of inflammatory fever under the head of symptomatic fever; and those of continued, or simple fever, under the heads of synochus, typhus mitior, or mixed fever, and, lastly, of typhus gravior," he makes a few remarks upon the symptoms and treatment of continued inflammatory fever, which contain no particular information; adds three cases of gastritis, hepatitis and pneumonia, by way of illustration, relates the appearances on dissection of two horses which died of inflammation in the chest, "as a link to connect inflammatory with typhoid fevers," and then passes on to the consideration of his second class.

"I consider typhoid or adynamic Fever to be generally symptomatic of morbid changes in the physical characters of the blood, and have as on former occasions, stated what those morbid changes are, viz. 'the crassamentum is dissolved, or broken into fragments, tinging the serum with its colour, which is sometimes of a very dark brown, and sometimes of a greenish hue:' but I have arranged inflammation under the head of symptomatic Fever, merely because it is more usually connected with some change in the structure of parts, discoverable after death; on the other hand, typhus Fever is connected with morbid changes, that primarily take place in the fluids, and produce morbid actions, and sometimes permanent changes of structure in the solid parts. These changes too, in the condition of the blood are distinguishable from those which we have stated to occur in inflammation; and the morbid actions excited relatively by these changes in the blood, are also distinct. In inflammatory Fever on the one hand, increased action; in typhoid Fevers on the other, debility, is almost the immediate consequence. On account of this debility being an essential character of typhoid Fevers, I denominated them adynamic.

"The following cases of typhoid Fever will be arranged under the separate heads of synochus or mixed Fever, typhus mitior, and typhus gravior—to the various degrees of which, simple Fever may be said to bear the same relation that *cynanche tonsillaris* does to *cynanche maligna*—*variola mitis et distincta* to *variola confluens et maligna*—or *diarrhœa simplex* to *dysenteria typhoidea*." 74.

A knowledge of the seat of fever involves much that may be known of its nature; and, therefore, we have, on the appearance of almost every new work, to canvass the merits of a new theory. But of the various solutions of this question which have been proposed, none has met with more advocates than that which is derived from the Humoral pathology; and it is undoubted, that few can more easily, or more effectually illustrate the complicated phenomena of this disease. We do regard it, however, as premature to advance and advocate any theory of fever grounded upon this system. Our acquaintance with the animal fluids is exceedingly imperfect; their constitution, even in a state of health, is unsatisfactorily made out; and the chemical alterations, which they suffer by disease, are almost entirely unknown. More especially in fever does this remark hold good; for, although there is not a single secretion in the system which is not soon and seriously disordered, not a single secretion has been analyzed with any chemical accuracy. How then, do we ask, can any experimental philosopher, pretending to any share of science, fabricate principles out of such uncertain materials, and upon these principles ingraft a practice, to which the lives of thousands are to be subjected? Such *kite-flying* is unworthy a genuine friend of the profession, and, without making any specific reference to the author who now happens to be before us, would we earnestly recommend every son of poetry and romance in medicine, if he must write, to give his speculations to the world in a separate form, unmixed with practical advice; and under some such honest title as *Raw Material;* that the reader may select, according to his own taste, facts or fancies, the chivalries of romance, or the calculations of reason. Were some such plan as this adopted, medicine might travel onward to the dignity of science, and, at all events, our libraries might be ultimately extricated out of their present confusion, and beneficially arranged into *poetry* and *prose*.

The water transpired by the skin and exhaled by the lungs, the urine and the bile, the secretions of the mouth and the excretions of the intestines, are equally changed in quantity and quality; and, while such alterations appear natural succedanea to a diseased condition of the blood, we have not one fact to prove, not one foundation-stone whereon to build the assertion, that the blood is the *una sedes et sola morbi.* By admitting, as we have done above, that the humoral pathology when applied to fever can account for many of its phenomena, we must be understood, therefore, to speak *comparatively;* for it is our present conviction, but which for the reasons already given we mean not to dignify with the formalities of a propounded theory, that the febrile poison, whatever it may be and wherever it may come from, acts primarily and principally upon the nervous system, and that the blood is, perhaps, the second animal constituent which is brought under its operation. This view is strongly supported by a host of facts, some of which only it will in the present place be necessary to adduce.

Were the blood the first habitation of fever, the blood should be the first to exhibit some morbid change; but the nervous system is the first which *visibly* suffers, nervous symptoms being the first which palpably appear. If our readers will take the trouble of turning up the first volume of the Dublin Hospital Reports, they will find what we deem a sufficient number of facts in behalf of this position. Dr. Marsh has, in a very able paper upon contagion, brought forward many cases of instantaneous seizure upon the application of the febrile poison; and in them all, nervous symptoms were the most severe and the first pronounced;—such as immediate loss of strength and spirits, general languor, coldness and pallidity of the surface, shivering, small weak pulse, headache, and pain of loins. Time was not allowed before the appearance of these symptoms for any serious alteration of the blood; and if any did occur, it could only have occurred gradually, so that the first symptoms should have been mild, and those which succeeded should have increased in severity, as the whole circulating mass became involved.* But in these cases, and how many of a similar kind are met with in every day's practice, disease, and not merely so, but the *whole disease* immediately supervened on a state of health, the date of attack was definite, and the whole system was overpowered in a moment. To us, consequently, it appears inexplicable by the symptoms and inconsistent with physiology, to limit in such cases the seat of fever to the blood, and we have selected these merely because, being strikingly marked in their origin, any reasonings founded upon them must be less obnoxious to fallacy. Prostration of strength and mental impotency are ever foremost in the van of febrile phenomena, and both are nought but conditions of the nervous system. The blood does not lose its ordinary physical characters until some time *after their appearance*; for when drawn immediately they appear, no appreciable alteration can be discovered in it, and this loss of external character, when it does occur, is always in a direct ratio to the intensity of the nervous symptoms; proving that disease of the blood in fever is not only subsequent in succession, but proportional in degree to derangement of the nervous system.

To propose a question to the Humoralists;—where, do we ask, have we any instance to which their doctrines can at all apply, in which *a whole disease involves a whole system in the space of a few minutes?* In purpura hæmorrhagica and scurvy, which are admitted to be the firmest fulcra of the Humoral pathology, their approach is slow and their course is gradual; the progress of the symptoms is in strict etiological keeping with the progress of the disease, less gives way to more and more to greater, and it is not, until the entire mass of blood is changed and the change is entire, that constitutional signs are manifested. Like some other poisons, that, which occasions fever seems to act directly upon the brain, spinal chord and nerves; or, in other language, upon the seat of life; its action, as well as theirs, will, of necessity, be modified by its strength and dose; when largely diluted, or acting upon a vigorous constitution, its latent period may be long, or its symptoms may be mild; when much concentrated, or acting upon a weak constitution, its introduction and action may be nearly cotemporaneous, and its symptoms intensely severe; and when its quantity and quality are both moderate, its period of latency and its symptoms will be proportionally modified. Hence it is that in India, where the poison is intense and the constitution enervated, a few hours only elapse between exposure and dissolution, and a few minutes only between the approach of symptoms and the period of exposure; and hence is it that in Europe, we have fever in all its types and degrees, and that a more careful ventilation

---

* The innumerable instances of deadly fevers being kindled up in the course of a few hours after exposure to concentrated malaria in the East and West Indies, as well as in several parts of Europe, prove that the nervous system is the first to feel the influence of the cause of fever.—Ed.

and a stricter system of cleanliness have so debilitated this plague, that in the present day it is not to be compared, either in frequency or fatality, with what it is described to have been some centuries ago.

"Although the first symptoms of typhoid or adynamic Fever, such as nausea, oppressed or hurried breathing, sighing, oscitation, and similar signs of debility are probably the consequence of the first impressions made by the exciting cause, be that impure air, malaria, or contagion; yet even these symptoms, as well as those consequent on them, may afterwards be fairly accounted for, by the diminished vitality of the blood, which is exhibited in the signs of its unhealthy condition, which have been already stated to take place. For blood, thus unfitted for the purpose it was intended for in the animal œconomy, is not only incapable of supporting healthy functions, but must tend to disturb them. That part of it sent to the brain, therefore, instead of promoting nervous energy, rather tends to overwhelm it. The same may be said of all the other functions which depend on the nervous energy of the brain, which alike depend on the vitality of the blood itself. Hence, muscular power, respiration, circulation, and all the secretions and excretions, are liable to be impeded or disturbed, in proportion to changes from the natural and healthy condition of the blood, which indicate diminution of its vitality.

"The animal functions are most strikingly affected by the diminution of nervous energy and vital influence which occurs in typhus Fever. Hence arise muscular inactivity, sighing, debility of the heart itself, and slow circulation; and hence, too, the duskiness and yellowness of the skin, petechiæ, brown striped tongue, green and black vomiting, venous congestion of the brain, and coma, as we shall find, when we come to consider those symptoms, more particularly." 101.

This train of inferences has nothing to support it but consistency with the author's peculiar doctrines, and as we have endeavoured to prove that several of these doctrines are scarcely more than hypothetical, the deductions extracted from them must be meagerly supported. Understanding malaria in its etymological and most extensive meaning, as nothing but foul air, it matters not how or where generated, there can be little doubt that it is by far the most operative cause in the production of fever, and that the blood sustains a full share of its deteriorating power. But it is as illogical, as it is unphysiological, for the Doctor to maintain that while, according to his own shewing, the first symptoms are nervous, the first animal constituent affected is the blood. We repeat it, that the blood exhibits no physical symptoms of disease, and the Doctor has not discovered one chemical symptom, or if he have he has declined publishing it, until after nervous symptoms have appeared. It coagulates as rapidly and separates as freely into serum and crassamentum before this period as during health; it appears neither softened nor dissolved; neither changed in color nor altered in proportion. All its alterations gradually appear as nervous symptoms are developed; increase with their confirmation and vanish with their removal; and, what to some may prove still more conclusive, many cases commence, progress, and terminate, without exhibiting through their whole course any such change in the circulating medium as the author describes. This is particularly true in the synochus of Loudon. In many instances have we examined blood in every stage of fever, and have we found it—dissolved, half coagulated and unseparated, like to a mass of jelly—no; but firm, duly separated, and carrying an inflamed surface, differing in no respect from that formed during common inflammation. In a great majority of cases it is otherwise we admit, but the scholastic dogma—exceptio probat regulam—will not here apply, and one instance of genuine continued fever occurring without any peculiar change in the blood disables the doctrine, and renders the humoral pathology as inapplicable to fever as it is to two-thirds of our ordinary diseases.

In continuation and support of the same theory the experiments of Prevost and Dumas, on the nature and action of the blood, and of Magendie, Barry, and Gaspard on the changes it undergoes by the introduction of certain fluids into the circulation, are brought forward and commented on; but, if the Doctor will excuse the candour of the observation, the citation of such authorities and facts we consider unfortunate, as both tend very strongly to support the views which it was his design to expose. In proof of this shall we present our readers with one of Gaspard's experiments, as detailed in the Journal de Physiologie, tome 2d, p. 16, and when we premise that this experiment has been selected as a favourite voucher by the Doctor out of many of a similar kind, no suspicion can attach to us of having suborned our witness.

"On the 14th of July, 1821, I injected into the right jugular of a moderately sized dog ozs. ijss. of fetid fluid, thick, but not acid, which was obtained by fermenting for two days cabbage leaves in water, of the temperature 20 degrees R. During the operation the animal made several efforts to swallow, vomited repeatedly, and became faint; some hours afterwards it appeared very uneasy, its abdomen was painful under pressure, its respiration was embarrassed, difficult and plaintive, and other pneumonic symptoms appeared. At the end of nine hours a very copious and fetid stool was passed, which was black as soot, and consisted of feces, mucus and putrid blood, and shortly after a second of a similar nature was voided. 15*th*. Faintness more considerable, adynamia, step faultering, pulse feeble, thirst excessive, respiration weak. But what appeared most remarkable was the irregular action of the heart, which beat at intervals with such force and noise as characterize the last stage of aneurism, or hypertrophy. During the next two days all the symptoms were moderated, but on the 18*th*. debility became extreme, gait tottering, thirst excessive, eyes red, inflamed, and covered with mucus, nostrils tumid and stuffed, mucous lining of mouth very red and inflamed. About noon a stool was passed, which contained some clots of blood, and had both the odour, consistence, and appearance of pus, and at night the animal expired;—five days after the operation.

*Inspection*. Skin, subcutaneous tissue and muscles presented the same dark appearance as after death from asphyxia, and were not altogether exempt from inflammation; conjunctivæ and lining membrane of the mouth of a deep red, or violet color, and coated with a film of mucus. The lungs were slightly inflamed; there were several brown patches upon the left ventricle of the heart, which resembled spots of ecchymosis, and were not confined to its surface, but had made some progress into its substance. The right ventricle was *en partie rempli* with a large fibro-albuminous concretion, hard, of a yellowish white color like fat, weighing two grains and a half, and adhering to the ventricle by a point of surface not broader than a nail, which seemed inflamed and ragged. This concretion extended into the pulmonary artery, both cavæ, vena azygos, and even the axillary and right jugular veins. It was of the same color and consistence throughout, and *was, probably, the cause of the palpitations which were so remarkable during life*. The œsophagus and stomach appeared sound, but the mucous membrane of the intestines, especially of the duodenum, rectum and some of the small intestines, was of a red violet color and inflamed, but this inflammation was unaccompanied by thickening, or ulceration, and resembled very much the effects of ecchymosis. The internal membrane of the rectum was still more affected, and its mucous glands were much enlarged and very visible. This intestine contained puriform matter, similar to that voided in the last dejection; the rest of the alimentary canal contained mucosities of a greyish white color, and very thick consistence. The mesenteric glands were filled with blood, and much inflamed. The gall-bladder was mottled externally with brown and violet spots, and was filled with a black bile, thick and ropy, like melted pitch."

One clause of this translation we have characterized with italics, as peculiarly remarkable. The fibro-albuminous substance here described seems to be nothing more than coagulated lymph, blocking up the large vessels of the heart, which the ancients were accustomed to designate by the fanciful name—*polypus*, and which is now every day met with on dissection. That this concretion could have existed to the extent and size above described for four days before death, or during no less than four-fourths of the time occupied by the experiment, is to us as marvellous as, we presume, it will be to our readers. The operation was performed on the 14th, and the palpitations, which are ascribed to the presence of this concretion, are noticed the next day, so that this concretion must have formed a few hours after the operation, and must have existed for four days after it. We are induced to notice this circumstance thus particularly for two reasons;—to prove that the palpitations, here ascribed to what was merely a post vitam product, must have depended on some other cause, and that the existence of this concretion is *toto cœlo* incompatible with Doctor Stoker's theory. If the blood be the seat of fever, if the change effected on the blood by fever be a dissolution of its natural consistence, and an intermixture of its separate constituents, and if the essence of fever be debility, here in a dog, evidently labouring under fever, as is proved by the symptoms, progress and pathology of his case, is a large deposit of coagulable lymph in the right side and large vessels of the heart; but coagulable lymph is neither the effect of dissolution, nor intermixture of the circulating medium, but of inflammation, or vascular activity. Supposing, however, this concretion to have been formed on the day succeeding the operation, that the circulation could proceed for four days with the right vessels and ventricle of the heart blocked up with lymph, and that the palpitation and vascular disturbance are to be ascribed to it, to what shall we ascribe the other and preceding symptoms? Whence arose the faintness and nausea and spasm of the œsophagus and vomiting, all of which occurred even during the performance of the operation? Will it be argued that these phenomena arose from the blood being poisoned, before the injected fluid could have vitiated one fourth of the mass, and before one drop of what had been vitiated could have reached the brain? The fetid liquid was thrown, not into the carotid artery, but into the jugular vein, so that an entire circulation was necessary ere the poison could operate *via circulationis* upon the cerebral system. The faintness, nausea, vomiting and spasms of the œsophagus during the operation, and the palpitation of the heart, the inefficient action of the lungs, the tottering strength, the vacillating step, and feeble pulse after it, are, consequently, to be esteemed primarily and purely nervous symptoms, as arising from and continued by nervous depression, and as completely independent, in the first instance, of any condition of the blood, as are the very similar phenomena occasioned by the injection of hydrocyanic acid. If water, weakly charged with this poison, be injected in very small quantity into the jugular vein of a dog, symptoms almost precisely the same with the former will be produced, and the stronger the injection which is used the more intense and immediate its effects; so that if the undiluted acid be alone employed, and it matters not whether the jugular vein or carotid artery be the channel chosen for the experiment, life may be extinguished as by a stroke of lightning; thus leaving no doubt as to the system upon which it operates, and at the same time furnishing an explanation of the comparative slowness of its action when employed in a diluted state.

To draw our observations on this part of the subject to a close, we conclude that there is not sufficient evidence afforded by nature to prove that the blood is the seat of fever—that its causes and symptoms, progress and treatment, terminations and pathology discountenance such a doctrine—that the author's arguments can be easily answered in accordance with a different theory—and that the stumbling-block, which he and every adherent of his school infallibly falls upon in investigating this question, is the mistaking a *secondary* for a *primary* effect. That the blood is generally changed in fever no one can and no one does deny, and that so soon as it does change every function and secretion must change along with it, every one admits; that this change consists in a diminished vitality, or partial death of the blood, those who believe in the life of this fluid, will feel little difficulty in conceding, and that the action of a peculiar poison is the producing cause of this change, all Fluidists and Solidists agree; but the Humoralists may believe and the Solidists may concede all these points, without attaching any thing beyond a subordinate influence to the state and action of the nervous system. And here lies the point of difference at which these sectarians stand at issue. The nervous system is the key-stone in the doctrinal fabric of the one party, the blood is that of the other; and while much of the creed of the latter is adopted and advocated by the former, the views of the Solidists take their rise from a very different source, and ultimately terminate in a very different result. It is the paramount importance of this result which has beguiled us into such a lengthened canvass of the opinions of Doctor Stoker, and we plead no other apology, either before the writer whom we are examining, or the reader whom we are addressing, for the size and spirit of this criticism. If it can be established that the blood is the habitation of this disease, on the derangement of which hangs every symptom, and on the measure of which derangement depends every danger; if it can be further shewn that the deranging cause is a debilitating poison, that the derangement wrought is neither more nor less than a partial disorganization and death of an organized and living fluid, whereon the very being of the animal system rests, and that a change in the qualities, not in the quantity of the blood, should be the object of our treatment, it is tolerably obvious that all active measures must for ever be abandoned, that the bloodvessel system might be wholly drained without affording relief, and that those, who have hitherto been inculcating and practising depletion, have been contending with nature. Were all this made out it were clear, indeed, that fever and debility are tolerably synonimous, that debilitating measures must, consequently, be fatal, that wine and bark are our surest remedies, leeches and the lancet our most dreaded foes. A doctrine which leads to inferences so practical cannot be loosely passed over, and an author, especially an author of Doctor Stoker's growth, who teaches such a doctrine, should be well prepared with evidence ere he deliberately gave it birth. The following quotation will supply us with an epitome of the treatment taught by this school, and with a specimen of the modest claims which it advances to catholicity;—

"The views taken, both of the nature and treatment of Fever, by Dr. Burne, entirely accord with those which may be found stated in my medical reports from the Fever Hospital, as well as in my separate Essays on that subject. And as

(when speaking of his denomination of Fever) I have already remarked, this leaves I think, no reasonable doubt of the epidemic Fevers of London, having lately become more typhoid or adynamic, than they had formerly been. It is further satisfactory to me to find, that the treatment which I had long since adopted and recommended in our typhoid Fevers, has been found suitable to the prevention and cure of those in London; and that, too, in proportion as they have acquired more of that form, with which I was best acquainted.

"The first principles of my practice thus appearing to be as generally received as they had before been rejected, it only remains for me to detail the remedies which I have employed; and with respect to these, too, I might be even more brief, from having little to add to the list of remedies in typhoid Fevers, which may be found at the 18th page of my 'Treatise on Fever,' published in London, A.D. 1814, as well as in my 'Medical Reports from the Cork-street Hospital;' but, that, as these publications may not be in the hands of the reader, some recapitulation of those remedies themselves; and my reasons for recommending them, supported as I have been by all my subsequent experience, may be permitted. They may be arranged according to their relative importance in the treatment of Fever, in the following order, viz.

| "IN MIXED FEVER. | IN TYPHOID FEVER. |
|---|---|
| Cleanliness. | Yeast or Barm. |
| Ventilation. | Wine. |
| Cool Regimen. | Aperients |
| Plentiful Dilution. | Emetics. |
| Purgatives. | Blisters. |
| Topical Bleeding. | Tepid, or Cold Affusion. |
| Antimonial, or James's Powder. | Peruvian Bark |

"Many other remedies may, no doubt, be occasionally employed with advantage for the relief of the symptoms which accompany peculiar forms of epidemics, or such as are produced by extraordinary idiosyncrasies. But these, according to my experience, are more frequently applicable than any others, in the treatment of our common indigenous Fever.

"Of the beneficial effects of the four first articles of this list of remedies in the treatment of Fever, it is no longer necessary to insist on, for they are no longer denied. For the same reasons, too, I need not, as on former occasions, enter more fully into explanation, why blood-letting has not a place in this list. When, however, I come to speak of topical bleeding, I shall have to state, that under the pestilential form which our epidemic Fevers have assumed since the year 1823, I have found it advisable to employ even this partial evacuation more sparingly and cautiously than in my first publications on Fever, I felt justified in recommending.

"With respect to Peruvian bark too, which I have here added to the list of remedies for typhoid Fevers; although, on former occasions, I stated, that 'I had not found it necessary for the cure of the continued Fevers in Dublin,' I have now to observe, that, under the growing malignity of these distempers, I have employed some preparations, especially the sulphate of quina, with obvious advantage, even in cases which did not partake of tendencies to remittent or intermittent forms; in such tendencies, however, the usefulness of that remedy was most manifest." 113.

We have met with much empty bravado in the chronicles of foppery, and we have occasionally read something very like it in the annals of heroism, but never, before the present moment, do we recollect to have seen a victory so triumphantly laid claim to by a few disabled survivors of a faction. There is an instance of a Grecian soldier, who, from a severe wound received during an engagement in which all his companions had been slain, was left apparently dead upon the field, but ultimately recovering from his insensibility, and observing that none of the opposite party could be seen, concluded that he was the sole survivor of the battle, instantly erected a trophy upon the spot on which he had lain, and claimed the victory. But our parable must here close; for shortly afterwards a few straggling soldiers came in sight, who had come out to plunder, he was immediately taken prisoner, and hearing that a large body of the enemy still survived, he *quietly* yielded up the victory which he had deemed his own. To be beaten is bad, but not to know when one has been beaten enough is inexcusable; a superiority either in strength, or number may occasion the one, but the other can only be attributed either to indifference, or despair. Doctor Stoker pays his London division of the faculty rather a doubtful compliment by citing Doctor Burne as their organ; for, without intending the slightest disrespect, we feel very confident that on a subject of so much importance, and we will say so imperfectly understood, this metropolis would scarcely elect him to be its representative. The names of Bateman, of Armstrong, and Clutterbuck, cannot be so soon forgotten; their works, which we feel no scruple in distinguishing as *sine comparatione* the most accurate and the most able, which have ever appeared on this disease in this or any other country, are not yet obsolete; the influence, which they have wielded over the faculty, has not yet been forfeited; and the portraits, which they draw, the FACULTY, at least in this section of the empire, continues to recognize as upon the whole faithfully taken and elegantly executed. The doctrine of localism has, perhaps, been adopted too exclusively by one, and treatment by depletion has, perhaps, been recommended too indiscriminately by another; but these are *peccata virtuti propinqua*—not so much faults as failings—and, without giving any sanction, in these philo-catholic times, to Popish theology, they cannot be far misplaced by being included in the class of *venial sins.*

In the preceding therapeutical catalogues the lancet is not once mentioned, only in one of them is local bleeding noticed, and save one it stands the last item upon the list! The peculiarity of this arrangement would not have been so striking, had the treatment proposed been intended for the lowest type of fever; but the Doctor's practice is as general, as universal as his principles, and in a second column a new bill of fare is drawn out adapted to adynamic typhus, at the head of which stand yeast and wine, and from which are entirely excluded both local and general bleeding. So that the first column, which excludes general bleeding, is intended for the cure of mixed fever—synochus—or the common continued fever of this country; and the second column, which excludes all bleeding, is intended for typhus, typhus mitior, and typhus gravior. This is, verily, a distinguished exemplification of the popular and reproachful adage—doctors differ,—and a sad illustration of the unharmonizing character of all medical opinion, and the capricious fickleness of all medical practice. But the above extract has one quality, nay two of truth—simplicity and universality; for nothing can be more easily understood than that we are not to bleed generally in *any* fever, and that we are not to bleed at all in those of the *weakest constitution;* and when it is added that these directions apply to every case, constitution, and circumstance, they are not less universal than easily comprehended. Besides, they are in strict keeping with the Doctor's other principles; for he, who has faith enough to believe his views as to the nature of fever, will generally have candour enough to carry them along with him into actual operation. Weakness calls for wine, as certainly as plethora admits of depletion; and the system, which is sinking from debility, stands the best chance of being saved by tonics. All this is strict

Baconian philosophy; rigidly orthodox deduction. Locke could not have extracted from such premises a more legitimate inference, and our admission of the inference cannot be optional, if the premises have been indisputably proved. These premises have already been analyzed, and arguments and facts, we hope sufficient both in strength and number, have been brought forward to show that, at least, they should not be implicitly received. The blood is changed in fever, but it is not the first part of the animal system which is changed, and it is, therefore, not the part which is entitled to the first attention. To treat fever, consequently, as a disease of the blood is to mistake its seat; to treat it as a disease of unmixed debility is to mistake its nature; and to recommend bark and antiseptics to counteract putrescency, and wine and nourishment to remove debility, is to physic effects in place of causes, to filter the stream while the fountain is left unpurified and unimproved.

If symptoms during life and appearances after death can speak any language, or mean any thing, it is that there is scarcely *among us* at the present period a single case of what has been called *simple* fever, some one organ being unproportionally affected, and very few cases which can be honestly called *ab ovo adynamic* fever. To talk of pure adynamic fever, of pure unmixed debility, of a disease made up almost exclusively of negatives, is to talk of a very rare occurrence, we had almost said of a very phantom. Such a phenomenon must be looked for in the tomes of the ancients, or in Dublin. It is an exotic to be travelled for, and any description of it, with which we may be favoured, can scarcely be turned in any other way by us to practical account, than by having it placed for exhibition in Great Russel Street, or Exeter Change. This may be suspected to savour of strong language, and so it does; but we are now contending on a strong point, with a strong antagonist;—the lives of thousands are at stake, and modesty or mannerism in such an engagement would be perfectly out of place. When we find acute pain in the head, or strong pulse at the wrist, a burning skin and ardent thirst, can we give wine? when we find the lungs gorged and labouring, can we give bark; when the find the heart active and the powers of life strong, can we dread weakness; and when we find improvement in every symptom following depletion in every form, can we execrate the lancet and prescribe stimuli? When we find upon dissection the most unequivocal vestiges of inflammation, how can we regard fever as unmixed debility, and how can we cure it with wine? Is pleurisy to be cured with wine? Is inflammation of the bowels or the brain to be cured with bark? Does excessive vascularity, or pus, or serum, or false membrane, or ulceration denote weakness? If thickening of membrane, effusion of lymph, collections of pus, ulcerations, adhesions, and consolidations bespeak not activity we know not what they indicate; if they be not german to inflammation we are ignorant of what inflammation is. Yet inflammation and ulceration of the mucous membrane of the intestines, inflammation and thickening of the mucous lining of the bronchiæ, inflammation and hepatization of the parenchyma of the lungs, inflammation, thickening, opacity, and adhesions of the membranes of the brain and spinal cord, increased vascularity, softening or hardening of the cerebral substance, and deposits of lymph, serum, and pus between the membranes, within the ventricles, and upon the base of the scull, are the ordinary appearances discovered by the scalpel in the bodies of those who have sunk under fever. Such appearances, if any thing at all be known of the phlegmasiæ, must be ascribed to unnatural vascular activity, and such increased action as can induce these appearances is surely not to be put down with wine. A firm and even a hard pulse may be deceitful, severe pain may prove equivocal, the common *rudicia* of augmented action may lead astray, and even a reduction of this pulse and pain by bleeding, and a removal of the other signs of augmented action by other depletory measures may still be obnoxious to error; but when to these symptomatic and curative expositions be added the necroscopic commentary, when the whole disease, both in its living and dead characters, is thus laid collectively before us, any ground for misconception upon a point like the present seems to us totally inconceivable. The nature of the work illustrates the character of the agent, any obscurity which may have attached to the language of the symptoms during life, receives its explanation in the appearances after death; so that while some plea may be urged by a practitioner for being a Brunonian during the life of his patient, none can possibly be advanced for remaining one after his dissolution.

The force of these arguments the Doctor endeavours to avoid, by citing the authority of two very distinguished anatomists against the occurrence of such pathological appearances after fever.

"Neither has morbid anatomy appeared to me to warrant the conclusions of those who hold the opinions of typhous fever being an essentially inflammatory disease. In some instances, I have observed the same partial turgescence of vessels which dissectors report, and likewise signs of inflammatory action in various parts of the bodies of those who died of fever; the former, however, by no means designated previous inflammation; and I could generally trace the commencement of inflammatory action, which led to the latter appearance, from local diseases, that preceded or supervened on fever, and sometimes at late stages; but in several cases, where I had witnessed the highest degree of febrile excitement before death, no such signs of turgescence, or of inflammation, were observable on dissection.

"In support of such statements, I was happy to be able to add, in the same Report, the satisfactory testimony of two of our most distinguished Anatomists. And besides their unquestionable qualifications, and numerous opportunities for examining the bodies brought to their several theatres for dissection, their opinions on anatomy have peculiar advantages of being deduced from observations that were not influenced by preconceived theory, or by cases selected for particular views.

"'In the midst,' Mr. Kirby writes in answer to my queries on the subject, 'of all the discussions relative to topical congestion, it was impossible however not to remark the singular want of accordance between the prevalent opinions and local appearances. The brain, so constantly supposed to be the seat of inflammation, rarely exhibited the characters indicative of such a state. In some instances this organ was much paler than usual; in a very few, amongst a great number of dissections, was there any evidence of sanguineous or serous effusion.' And after recounting his observations on all the other vital organs, he thus concludes:—'In short, in a great majority of cases, so little did any particular organ seem to suffer, that I have wondered what could have been the cause of death.'

"With the foregoing statement, the answers of Dr. Macartney, Anatomical Professor of Trinity College, Dublin, to the subsequent queries of Dr. Barker on the same subject, are coincident, and appear to me further to corroborate the opinion, that typhous and inflammatory fevers are distinct. I may, therefore, be allowed to add the following extract from Dr. Barker's Report for the Years 1817 and 1818, as published in the second volume of Transactions of the Irish College of Physicians, *page* 574.

"'He, (Dr. Macartney) informs me, that having reviewed his notes on the

'anatomical examination of persons who died of typhous fever, he can state, as the 'result of his experience, that morbid appearances in typhous fever are not those 'of common visceral inflammation.' And having stated more fully the actual observations on which this opinion was founded, he concludes thus:—

" 'Two facts deserve to be recollected: first, that the duration of general fever 'and visceral inflammation are not the same,—second, that internal inflammations 'are very common in hot-blooded animals, but idiopathic fever is peculiar to the 'human kind.' It may be added, that processes of an inflammatory nature are fitted for repairing parts that have their functions interrupted, or their structure injured, but the effects of typhous fever have no such power." cxiv.

This passage has been chosen, because it is the most favourable to the Doctor's views on this point of any in the book; and when it is considered, that a subject of so much importance as the pathology of fever is passed over so lamely, at the very time the writer is labouring to prove that fever has nothing inflammatory in its composition, the haste with which this part of the subject has been got rid of must appear somewhat singular. It may have been maintained in a moment of rash and thoughtless dogmatizing, that fever is a disease as *purely* inflammatory as cephalitis or pneumonia, and ground may have been given to the followers of Brown to presume upon the same pathological appearances; but as such an *ultra* doctrine can be held by few in the present day, any arguments tending to subvert it are quite unnecessary. Clutterbuck was once accused of teaching it; but, if the accusation were ever just, we believe there no longer remains any pretext for its continuance. At all events, such a sentiment has never been advocated by us, and we totally repudiate the charge that it is the recognized doctrine of the Solidists. Fever is inflammatory, but it is not purely inflammatory; erysipelas is inflammatory, but it is not pure inflammation. Typhus and synocha are mingled together in different doses in the common fever of this country; there is vascular activity with nervous depression, strength with weakness, and it is just the varying ratios, which these two qualities maintain towards each other, that supply us with all the varying types and shadows of this disease. If the inflammatory quality preponderate the disease will be marked by activity, if the nervous it will be characterized by debility; if the inflammatory symptoms be severe the cotemporaneous nervous symptoms will be moderate, if the nervous symptoms be severe the inflammatory symptoms will be less distinct; and thus, by a mere interchange of the ratios between these two classes of symptoms, may we manufacture continued fever of every character, and construct a *pyrotological scale*, the upper half of which shall consist of fevers *plus* or positively inflammatory, the lower half of fevers *plus* or positively nervous. The admixture of nervous with inflammatory action can be conceived so slight and disproportioned, that the disease may amount to the purest form of synocha; and there may be such an excess of nervous action as shall nearly, or altogether disable the phlogistic quality, and produce a fever of the very lowest imaginable type. But in every case and type of fever there are two *constituentia*, equally essential to its existence, and the proportions which these bear to each other being given, it will be easy to discover the nosological position of every case we meet with.

The phlogistic appearances revealed by the scalpel after fever cannot be the same with those observed in pure and unadulterated inflammation. They will be modified in *kind* by the nervous action with which the inflammation is complicated, and they will vary in *degree* according to the relation which obtained between the doses of the febrile elements. The evidence of Dr. Macartney and Mr. Kirby has, consequently, no weight against us, as it attests a mere truism—a fact we are equally disposed to admit with these gentlemen;—on the contrary, in the assertion which it contains, that "morbid appearances in typhus fever are not those of common visceral inflammation," two admissions are comprehended, co-extensive with the doctrines we are arguing for:—1st, that the morbid appearances are inflammatory; and, 2dly, that these appearances are different from those of common inflammation. These two admissions are made in this extract, the second directly and the first constructively, and in these two admissions are contained the identical views which it has been our object to defend. As to the author's declaration, that in several cases where he had witnessed the *highest degree of febrile excitement* before death "no *such* signs of inflammation or turgescence were observable on dissection," we have only to remark that it is quite at antipodes to our experience; and when we are told that *processes of an inflammatory nature are fitted for repairing parts that have their functions interrupted, or their structure injured: while the effects of typhous fever have no such power*, we must reply that we scarcely understand the diction, and not at all the sense of the passage, either in a pathological or physiological point of view. Processes of an inflammatory nature can never be considered harmless and seldom safe; and to talk of them as calculated to repair injured structure and impaired function, is to talk of pneumonia as well calculated to cure hepatization of the lungs and gastritis as conducive to good digestion! Fever was once viewed as a therapeutical agent of the *vis medicatrix naturæ;* and, whether she purged her patient by inducing diarrhœa, or bled him by causing hæmorrhage; whether she worked through the kidneys or the skin, Nature was implicitly confided in, all that she did was deemed good and able practice, and a refreshing sleep, or a critical tremor was set down as different ways of arriving at the same end. Much of this is fancy and some of it is fact, but we are acquainted with no heresy that ever considered cephalitis as a salutary process; and, although the preservative powers of the system are excited by the approach of danger, and nature has recourse to every plan to avoid or counteract it, pneumonia must ever, we fear, be esteemed a wretched means of improving, either the structure, or the functions of the lungs. When the morbid cause has ceased to act, or when the constitution has strength enough to resist its influence, health may be restored; but it is only restored when the exciting cause and the unsalutary effects which it has occasioned are removed, and this is quite as true in common fever as in common inflammation. Ulceration of the intestines, inflammation of the bronchiæ, or of the arachnoid, and diseased secretions are *effects* of fever; yet they all can be and are removed by the preservative powers of the constitution, as effectually as enteritis, bronchitis, cephalitis, or any other *itis.* None of them certainly are salutary processes, either fitted or designed to repair diseased structure and function; they are morbid processes, calculated to disorder both; but so is it in ordinary inflammation, and a fractured cranium, or a rotten lung, might as justly be talked of as fitted to repair structure and function, as inflammation of the eye or brain.

We have now concluded our observations upon that part of Doctor Stoker's work which is devoted to fever, and it occupies seven-eighths of the *Medical Report;* and we feel considerably disappointed by having been compelled to differ from so respectable a writer so often and so widely, upon the very vitals of this most important disease; but where the interests of humanity are rendered, if possible, still more momentous by being bound up with the interests of science, truth must be maintained boldly, and error must be boldly opposed. Had Doctor Stoker circumscribed the applicability of his conclusions to Dublin, or to the Cork Street Hospital, the special theatre of his observations, however violently tending we might have been to scepticism, the character of the author would have procured respect for his principles, and, probably, some reverence for his practice. But when, uninvited and unprepared, he steps beyond the sphere of his own movements, and, leaving his own workshop, begins to cut and to carve after his own taste and his own models a foreign and unknown material, which moreover is the property of others, it cannot be deemed bold for the owner to look after the interests of his property, although by doing so he may, peradventure, expose the principles, or hurt the feelings of the intruder. When the conclusions which he draws at home, and which should be devoted to home consumption, are bound up and sent abroad; when the practice which he has himself chosen is indiscriminately recommended, and when it is his main and constant effort to stamp upon fever in the abstract the local features which it wears, or is supposed to wear in Dublin, we are goaded to the contest in self-defence, had we no other motive, and we are urged to repel a generalization, which can no more apply to the treatment of fever among us than it can to the treatment of syphilis in Dublin. There is no question but this disease occasionally assumes a very typhoid form, and the Doctor may have met with more than his proportion of this type; nay, it is certain, and more especially in Ireland for reasons already given in the commencement of this review, that whole epidemics have been characterized by debility; but these are the exceptions and not the rule, and it would be to subvert the very elements of common sense to transfer generic characters to a rare and almost expatriated species. Before fever, as a generic disease, can be described and treated as it is in this work, consistently with nature, the disease must change its seat, and alter its symptoms, and furnish a new series of pathological phenomena. Such revolutions, however improbable, are still possible; but to describe them now must either be conjectural or prophetic, and whether one or other, is wholly supererogatory, as there will be time and talent enough, we have little doubt, after their appearance, to announce their arrival.

In the remaining pages of this volume some remarks are made on intermittent fever, tic doloreux, measles, small-pox, and dropsy; but as the few observations which he makes are chiefly intended to illustrate Doctor Stoker's general views of pathology, and as we feel no inclination to extend at present our criticisms, we shall merely extract a few passages, and then close the review. After remarking that tertian agues prevailed in the Spring and quotidians in the Summer—that he seldom found it necessary to bleed, and when he did bleed, that he preferred the apyrexial period for the operation—that he generally confided the case to an emetic in the first instance, and to sulphate of quina afterwards, he then details three cases of tic doloreux, and adds;—

"I have introduced these cases of *tic doloreux* under the head of intermittent Fevers of the past year, because the production of these different forms of disease, appeared to me to be alike influenced by malaria, as their chief exciting cause; and as their natures were very similar, I treated them also in nearly a similar manner. In both ague and intermittent neuralgia, I believe the first impression of the exciting cause to be made on the gastric juices; and that the disordered function of digestion, and the consequent morbid condition of the chyle, and of the other contents of the stomach, whether ultimately absorbed, and carried into the sanguiferous system, or carried downwards by the primæ viæ, become in their transit, a chief source of all the succeeding symptoms; the diurnal revolutions of those symptoms, probably depending upon the habitual revolutions in the functions of digestion, produced by the custom of taking food at particular hours of the day. When these periodical diseases, however, become more established, it is probable, not only that the fluids are further affected, but that consequent changes are excited, in the texture of the vessels of the chyliferous, sanguiferous, or lymphatic systems, or even in the nerves themselves; and hence, the morbid condition of the fluids, may be *the primary source of organic disease.*

"On these principles perhaps, the efficacy of emetics, both in shortening the paroxysm of intermittent Fever or neuralgia, or in preparing for the successful exhibition of the different preparations of Peruvian bark, may be satisfactorily accounted for; and the same may be said with respect to the advantages which are sometimes derived from anodynes, previous to the exhibition of the bark; for, in such cases, the acrimony of the gastric juices, as will sometimes happen from mere dilution from warm water, may be so far obviated as not to excite morbid actions on the extremities, either of the vessels or the nerves." 181.

The tumours, which have been sometimes observed to disappear during the paroxysms of an ague, and the sudden enlargement of the liver and spleen which is occasionally witnessed in the intervals, are accounted for by the Doctor on the principle of congestion, or an unequal distribution of the fluids; and there are certainly many reasons for regarding this opinion as correct. The existence of congestive fever is disputed by many, but it is likely that the hesitation, which some feel in admitting this species, arises more from the manner in which the fact is described, than from any want of evidence in support of the fact itself. It was long ago remarked by Fordyce, that the greatest danger to be apprehended during fever was unequal action, or the occurrence of local congestion; and dissection has abundantly confirmed this remark. To understand congestion in its simplest and most useful sense, we believe there are few cases of fever which are not to a certain degree congestive; and while this fact may have been cramped, by limiting this species to such patients as have an anemiated, cold surface, and a weak, slow pulse, there is no reason why the fact itself should be controverted, nor why our practice should not benefit by the information it affords.

"When treating on the pathology of our common continued Fevers, I have stated the reasons which led me to suppose that of late years they have generally had a tendency to a typhoid character; and the same reasoning appears to me to be applicable to the exanthemata, which also have been epidemic at certain periods of the past year, an opinion which I shall illustrate by cases of *measles* and *small-pox*, and refer, with respect to *erysipelas*, and other of the exanthemata prevalent, to the reports, I have already published, of former years.

"MEASLES.

"Measles prevailed very generally during the months of January, March, April, May, and June, in the past year; and in many of the cases I met with, both in hospital and private practice, it was obstinate and severe, during the different stages, but, in every case, the applicability of bleeding in the eruptive stage, appeared to me to be the paramount question, both in importance and difficulty." 184.

The answer which this question received was unfavourable to bleeding; rubefacients applied to the chest, small doses of tartar emetic frequently repeated, and demulcent expectorants were preferred; but afterwards he adds:—

"In two cases, however, of the sequelæ of measles, one a male, and the other a female, I found it necessary to direct bleeding repeatedly for the relief of pulmonary affections, such as from the pernicious effects of the prevailing north-east winds, were then often to be met with. In all such cases, the blood was deeply and densely buffed, and the operation in general gave relief; relief, however, which was promoted by the exhibition of nauseating doses of the solution of tartar emetic, in aid of other more cordial expectorants, and of sudorifics, particularly preparations of nitre and ipecachuanha, and of ammonia. The recovery of these patients from the sequelæ of measles, was slow, but ultimately complete." 190.

Although vaccination is not an infallible preventive of variola, there can be no doubt that the varioloid eruption which occasionally succeeds it is much less dangerous than the unmodified disease; and it would also appear that the contagious poison, exhaled during small pox after vaccination, as well as the matter secreted by the pustules, will generate a much milder disease than the ordinary one, in those who are unprotected or have never been vaccinated.

"I might detail very satisfactory evidence indeed, of small-pox having extended by its proper contagion through numerous families, who had been previously vaccinated, and who had been deemed by eminent practitioners, quite secure from the danger of any such attack. In one of these families, in particular, I had been very intimate from the birth of the children, till they arrived at adult age, in 1827, when all, six in number, were successively attacked with the varioloid disease. I had visited them in all the diseases incidental to children, and was called on to witness the regular progress of the areola after vaccination, so that I was able to add my testimony to that of the several respectable medical practitioners, who had vaccinated them; yet the successive attacks of the eruptive Fever of the small-pox were in all until the seventh or ninth day, apparently as severe, as if no such immunity had been given; and in one of them, a young lady of seventeen years of age, the pustules were confluent, and succeeded by secondary Fever, which for many days had an alarming aspect, and was of doubtful issue. The sequel, too, marked the virulency of the preceding disease; for, not only considerable pitting over the face and bosom, after the desquamation of the cuticle, but also considerable delicacy both of the pulmonary and hepatic systems succeeded, and which only disappeared in the course of last summer. The pitting too has become less perceptible: so that it can now be discovered only on close examination. A similar pitting followed a similar attack in another young lady of the same age, whom I attended in the past year; she was remarkable, too, for the same delicacy of complexion: in this instance also, the marks of pitting are becoming gradually more faint, and probably will totally disappear.

"Of the six cases which were admitted with small-pox into the wards of the Hospital under my care, in the months of April and May, when the disease was most prevalent, four were females and two males; three of the females had been vaccinated, and the other inoculated with the small-pox; but neither of the males were provided with any such protection. All had arrived at adult age; but that of the eldest did not exceed thirty years. In three of the four females, the eruptive stage was severe, and of these three, she who had been inoculated with the small-pox, had the disease most severely until the ninth day; and in these three, the eruption was ultimately confluent, and pustular; so much so, that I deemed it advisable to puncture several of the largest pustules, in order to moderate the disease, as recommended by Tissot and Burserius; a practice that, in many instances of the worst forms of small-pox, I have found highly beneficial, both in moderating existing Fever, and preventing secondary Fever. In all these females, however, a remarkable decline of the symptoms was observed on the 9th day, and no pitting remained after desquamation."

In the first part of Doctor Stoker's Pathological Observations, published in 1823, and which will be found noticed in this Review for June, 1824, several principles are laid down and commented on regarding the nature and cure of dropsy. This disease, or rather class of diseases was then described under two divisions—dynamic and adynamic; the first admitted bleeding, the second did not, and both were made to illustrate the humoral pathology. A few *dynamic* cases are now added to those formerly given, and the principles of the old division continue to be retained. As there is nothing new, therefore, to encourage further notice, we shall here dismiss our author for the present; hoping ere long to have another opportunity, in perhaps another place, of taking up some pathological points which have been now of necessity omitted, and of still more minutely analyzing the principles which we have found it our duty to criticise. And we rest satisfied, from our general knowledge of the author, that difference of sentiment between members of the same profession, who can have no other motive for their difference than a sincere regard for the interests of their common profession, will never give rise to unpleasant feeling in either. We believe that Doctor Stoker has been teaching certain doctrines injurious to this profession, and we are called upon to correct error, wherever, whenever, and however it may present itself; and knowing, as we do experimentally, the baneful influence of Brunonianism over the management of fever, it shall ever be our business, as it is our duty, to attack and expose it, no matter by what authority, or with what ingenuity it may be advocated.

